# An indication of current views of Australian general practitioners towards chiropractic and osteopathy: a cross-sectional study

**DOI:** 10.1186/s12998-016-0119-6

**Published:** 2016-11-01

**Authors:** Roger M. Engel, Robyn Beirman, Sandra Grace

**Affiliations:** 1 Department of Chiropractic, Macquarie University, Sydney, NSW 2109 Australia; 2 School of Medical and Applied Sciences, Central Queensland University, Sydney, Australia; 3 School of Health & Human Sciences, Southern Cross University, Lismore, Australia

**Keywords:** Chiropractic, Osteopathy, General Practitioners, Complementary Medicine

## Abstract

**Background:**

While the role of complementary medicine therapies such as chiropractic and osteopathy is yet to be clearly delineated in the Australian context, demand for these services remains high. The attitudes of general practitioners towards chiropractors and osteopaths may have played a part in producing this outcome. However, this view is based on data that were more than 10 years old. Current anecdotal evidence suggests that the previous level of support may be declining in sections of the Australian medical profession. An assessment of the current views of general practitioners towards chiropractors and osteopaths is called for. The results being reported here represent the first stage of this assessment.

**Methods:**

This cross-sectional study was designed as a quantitative descriptive study using an anonymous online survey that included closed and open-ended questions with opportunities provided for free text. The target population was Australian general practitioners. Inclusion criteria included current medical registration, membership of the Royal Australian College of General Practitioners and currently practicing as a general practitioner in Australia. The data being reported here were collected between May and December, 2014.

**Results:**

There were 630 respondents to the online survey during this period representing a response rate of 2.6 %. Results were not uniform for the two professions. More general practitioners believed chiropractic education was not evidence-based compared to osteopathic education (70 % and 50 % respectively) while scope of practice was viewed as similar for both professions. A majority of general practitioners had never referred a patient to either profession (chiropractic: 60 %; osteopathy: 66 %) with approximately two-thirds not interested in learning more about their education (chiropractors: 68 %; osteopaths: 63 %).

**Conclusions:**

This study provides an indication of the current views of Australian general practitioners towards chiropractors and osteopaths. The findings suggest that attitudes may have become less favourable with a growing intolerance towards both professions. If confirmed, this has the potential to impact health service provision. The results from this cross-sectional study suggest that obtaining representative general practitioner views using online surveys is difficult and another approach is needed to supplement or replace the current recruitment strategy.'

**Electronic supplementary material:**

The online version of this article (doi:10.1186/s12998-016-0119-6) contains supplementary material, which is available to authorized users.

## Background

Complementary Medicine (CM) is a broad term used to describe a range of health care medicines, therapies and products not generally considered within the domain of conventional medicine [1]. Reports of non-evidence based CM being used in place of evidence-based treatments for patients with serious but treatable conditions continue to cause unease among Australian health authorities [1]. Other concerns about CM include their efficacy, interactions between medically prescribed treatments and financial cost to the community [1, 2].

While CM therapies such as chiropractic and osteopathy are formally recognised as allied health professions in Australia [3] their role within the healthcare system is yet to be clearly delineated. Accompanying this uncertainty are general concerns over safety [4] and education [5–7]. Specific issues such as the status of teaching programs within the Australian public university system continue to be raised despite the requirement that all osteopaths and chiropractors undertake five years of tertiary education in government accredited university programs in order to be licensed to practice.

Notwithstanding concerns about safety and quality of education, the majority of chiropractors and osteopaths appear to be acting in a responsible and reliable manner [8, 9]. According to the Australian Health Practitioner Regulation Agency (AHPRA), in the period 2014–2015 notifications for chiropractors accounted for 1.5 % of the registration base [8] and for osteopaths 0.7 % of the registration base [9].

Instances have occurred where practitioners behaved in a manner that was unacceptable and ‘out of step’ with current models of good practice. The views of a small group of chiropractors on the issue of vaccination or the use of ‘prolonged water-only fasting’ by an osteopath are examples of this [10, 11]. These examples reflect management of non-musculoskeletal conditions by chiropractors and osteopaths. It is possible that general medical practitioners’ (GPs) attitudes towards chiropractic and osteopathy differ according to whether they are managing musculoskeletal or non-musculoskeletal conditions. There is a small but emerging evidence base for the effectiveness of chiropractic and osteopathy in the management of musculoskeletal conditions like persistent lower back pain [12], although a Cochrane review in 2011 concluded that there is insufficient evidence to confirm or say whether they were more or less effective than conventional treatments [13]. Treatments for non-musculoskeletal conditions like asthma, dysmenorrhoea and infantile colic, however, have only limited or no scientific evidence to date [14, 15]. One of the issues with studies on the effectiveness of chiropractic and osteopathy is that many of them have been conducted on single treatments that fail to capture the complexity of chiropractic or osteopathic interventions [14, 15].

Despite the publicity generated by unacceptable behaviour of a few practitioners, the demand for chiropractic and osteopathic services continues to remain high [16–18] with some evidence to show that GP attitudes may have played a role in producing this outcome. Three separate studies conducted in 1997, 2000 and 2013, reported high levels of support for both chiropractic and osteopathic services from GPs in Victoria, Tasmania and rural and regional New South Wales [19–21]. A similar result was recorded in a national survey conducted in 2004. The authors of that study concluded that chiropractic was “truly complementary rather than alternative to conventional medicine and (could) be considered mainstream in Australian general practice” [2].

Reports of collaboration between chiropractors and medical practitioners have appeared in the scientific literature. In Canada and the UK, chiropractors work alongside medical practitioners in public hospitals and multi-disciplinary medical centres [22] while in Norway, high referral rates from GPs to chiropractors has been credited with improving patient outcomes and reducing costs associated with treating certain musculoskeletal conditions [23, 24].

In 2015, AHPRA reported that there were 4998 chiropractors and 2000 osteopaths registered in Australia [8, 9] and yet similar reports of collaboration between chiropractors or osteopaths and medical practitioners have not been recorded in the Australian arena. This represents a failure to translate the positive views held by GPs into clinical practice. One possible explanation for this failure may be that the views of GPs towards chiropractors and osteopaths have changed. In light of the absence of any large scale collaboration between the professions and the recent comments made by senior Australian academics doctors and scientists who labelled CM courses such as chiropractic as ‘non-science’ and ‘pseudo-science’ [25–27], it would be presumptuous to assume that the opinions expressed in 2004 were still current without producing some evidence to support such a claim. Furthermore, with an increase in public awareness about CM the views of GPs towards popular types of CM may influence the way they discuss other types of CM with their patients. This has the potential to impact the doctor-patient relationship as management strategies are becoming increasingly patient focused.

The aim of this study was to assess the current views of Australian GPs towards two types of CM: chiropractic and osteopathy. We adopted a two stage process with this manuscript reporting the results from stage one, a cross-sectional study designed to test the feasibility of a large-scale study, including the effectiveness of the recruitment strategy to achieve the required response rate and sample size and to produce an indication of the current national view. These results will be used to inform the design of stage two, a large-scale survey on the views of Australian GPs towards chiropractic and osteopathy.

## Methods

This cross-sectional study was designed as a quantitative descriptive study using an anonymous online survey. The questions were developed independently of any previous survey and tested on a focus group of 19 experienced GPs attending a regional training conference. These GPs were supervisors within a vocational training program for doctors wanting to specialise in general practice in New South Wales. The group comprised 11 females and 8 males and had an average age of 46 years. In response to feedback received from this group the language used in a number of the questions relating to both professions was modified and additional demographic questions added to the survey. The modified survey became the format used in this cross-sectional study.

The target population was Australian GPs currently working in private practice. Workforce statistics from 2013 to 2014 showed there were 32,401 GPs of which 24,377 were vocationally registered [28]. Inclusion criteria for the study were current Australian medical registration, membership of the Royal Australian College of General Practitioners (RACGP) and currently practising as a GP within Australia. Medical specialists, interns, residents and speciality registrars, non-practising practitioners and medical students were excluded from the study. The survey link was initially promoted at a national GP conference held in Sydney in 2014 followed by publication on the RACGP website and electronic publications and advertorials on a national commercial website for Australian doctors. The data being reported here were collected between May and December, 2014. These cut-off dates were chosen for convenience and not for any other reason.

The survey contained a total of 43 questions divided into sections that covered demographics (e.g. length of time in practice, postcode of practice, gender and age group), awareness of, and opinions about, chiropractic and osteopathy (e.g. source of information, first-hand experience, opinions about benefits, referral practices), knowledge of chiropractic and osteopathic education, scope of practice and communication patterns between GPs and chiropractors and osteopaths (see Additional file 1). Thirty-nine questions were structured as closed-ended questions with options provided. Two questions were open-ended and provided space for free text and two questions were mixed (i.e. options were provided plus the opportunity to add free text). Eighteen questions utilised a 5-point Likert-scale with response options ranging from strongly disagree to strongly agree.

Statistical analysis was performed on the results of pairs of questions relating to chiropractic and osteopathy using McNemar’s test for dependent binary data. Confidence intervals were calculated according to the method described by Agresti [29]. A level of *p* = 0.05 was set as the threshold for statistical significance. Questions involving binary responses (yes/no answer) were collapsed by grouping the ‘Maybe’, ‘Don’t remember’ and ‘Don’t know’ responses together with the ‘NO’ answers. Questions involving ordinal responses were collapsed in a similar manner. For questions about a respondent’s agreement with a given statement, the ‘Strongly agree’ and ‘Agree’ responses were combined under one group while the ‘Neither agree nor disagree’, ‘Disagree’ and ‘Strongly disagree’ responses were combined under another group. For questions relating to a respondent’s level of knowledge, the ‘Very knowledgeable’, ‘Knowledgeable’ and ‘Some knowledge’ responses were combined under one group while the ‘Not very much knowledge’ and ‘No knowledge’ responses were combined under another group. For questions about a respondent’s level of satisfaction with the methods of communication between each profession, the ‘Very satisfied’ and ‘Satisfied’ responses were combined under one group while the ‘Neither satisfied nor dissatisfied’, ‘Dissatisfied’, ‘Very dissatisfied’ and ‘Haven’t communicated enough to respond’ responses were combined under another group. For questions relating to where a respondent gets their information about each profession, direct comparison between professions was made for each of the given categories.

All respondents consented to participate in the online survey.

The study was approved by Macquarie University’s Human Research Ethics Committee (Approval number: 5201400200).

## Results

In total there were 630 respondents who met the inclusion criteria and responded to the online survey during the nominated period (see Additional file 2). This represented a response rate of 2.6 % (630 out of a total of 24,377). A comparison between the demographics of our respondents and the 2014 GP workforce data (see Table 1) show a reasonably good correlation for gender and location by state [30, 31]. As for age, a greater proportion of our respondents were over 40 years of age compared to the national average [30]. With respect to years in practice, a greater proportion of our respondents had less than 10 years in practice compared to the national average [32].

**Table 1:** Respondent characteristics (*n* = 630)*

Source of data								Non-responders
	Gender							20
	Male	Female						
Study	59	41						
Profession ^a^	60	40						
	Age (years)							15
	<30	30–40	41–50	51–60	>60			
Study	5	28	21	23	23			
	<30	30–39	40–49	50–59	>60			
Profession ^a^	25	48	14	7	6			
	Years in practice							7
	0–2	3–5	6–10	11–15	16–20	20+		
Study	10	14	15	10	6	45		
	<2	2–5	6–10	11–19		20+		
Profession ^b^	1	10	9	16		64		
	Geographical region by postcode							23
	NSW	Vic	Qld	SA	WA	Tas	ACT	
Study	32	23	24	7	9	3	2	
Profession ^c^	33	25	20	8	10	2	2	

* All figures given as % of responders; Figures for non-responders are number for each category; Study refers to results from the cross-sectional study; Profession refers to GP workforce data

^a^ Medical Board of Australia. Medical Practitioner Registrant Data: September 2014 http://www.medicalboard.gov.au/

^b^ University of Sydney. General practice activity in Australia 2013–14. http://ses.library.usyd.edu.au/bitstream/2123/11882/4/9781743324226_ONLINE.pdf

^c^ Australian Institute of Health & Welfare 2014. http://www.aihw.gov.au/workforce/medical/additional/

Responses to all of the closed-ended questions including the rate of non-responders are reported in Tables 2 and 3. Respondents who indicated that they had not communicated or interacted with chiropractors or osteopaths were directed to skip the questions about satisfaction with such communication or interaction. Only 30 % of GPs obtained information about chiropractic from medical journals while 20 % obtained information about osteopathy from the same source. More GPs disagreed with the statement that there was a growing body of evidence on the efficacy of chiropractic (66 %) compared to osteopathy (49 %). With respect to professional education in Australia, 70 % of GPs believed chiropractic education was not primarily evidence-based whereas only 50 % held the same view about osteopathic education. When asked, ‘Would a better understanding of chiropractic make you reconsider referring a patient to a chiropractor?’ 62 % of GPs responded in the negative while only 49 % did so for osteopathy and osteopaths. In response to the question ‘Would you co-manage a patient with a chiropractor if clinically appropriate?’ 32 % of GPs responded that they would while 33 % responded similarly for the same question about osteopaths. With respect to media coverage and how it may have affected a GP’s ability to understand the role of a chiropractor or osteopath, 29 % either ‘strongly agreed’ or ‘agreed’ that it did affect their understanding of chiropractic whereas only 15 % held the same view about media coverage and osteopathy.

**Table 2:** Difference in GP attitudes about Chiropractors and Osteopaths: binary response questions^a^ (*n* = 630)

Survey question	YES	NO^b^	Difference^c^ (95 % CI)	p	Non-responders^d^
Have you ever witnessed a treatment by a chiropractor?	304 (50)	305 (50)			21
Have you ever witnessed a treatment by an osteopath?	162 (27)	449 (73)	23 (19–28)	< 0.01*	19
Have you ever been a recipient of a treatment by a chiropractor?	164 (27)	445 (73)			21
Have you ever been a recipient of a treatment by an osteopath?	101 (17)	508 (83)	10 (7–14)	< 0.01*	21
Have you ever referred a patient to a chiropractor?	239 (40)	365 (60)			26
Have you ever referred a patient to an osteopath?	205 (34)	396 (66)	6 (1–10)	0.01*	29
Have you ever had a patient referred to you by a chiropractor?	297 (49)	306 (51)			27
Have you ever had a patient referred to you by an osteopath?	180 (30)	424 (70)	19 (15–24)	< 0.01*	26
Do you know any of the chiropractors in the area around your practice?	378 (64)	217 (36)			35
Do you know any of the osteopaths in the area around your practice?	178 (30)	410 (70)	34 (29–38)	< 0.01*	42
For which of the following conditions do you believe that treatment can be helpful?					
Tension headache (C)	163 (28)	412 (72)			55
Tension headache (O)	164 (28)	418 (72)	0 (−3–4)	0.79	48
Cervicogenic headache (C)	212 (37)	368 (63)			50
Cervicogenic headache (O)	182 (31)	403 (69)	6 (2–10)	0.01*	45
Migraine (C)	85 (15)	485 (85)			60
Migraine (O)	89 (16)	478 (84)	1 (−4–2)	0.54	63
Mechanical low back pain (C)	307 (53)	278 (47)			45
Mechanical low back pain (O)	244 (42)	342 (58)	11 (7–15)	< 0.01*	44
Facilitating mobility in patients with arthritides (C)	153 (27)	423 (73)			54
Facilitating mobility in patients with arthritides (O)	167 (29)	415 (71)	2 (−6–2)	0.44	48
Neck and upper back pain due to muscle tension (C)	225 (39)	352 (61)			53
Neck and upper back pain due to muscle tension (O)	217 (38)	360 (62)	1 (−3–7)	0.42	53
Would you co-manage a patient with a chiropractor (if clinically appropriate)?	189 (32)	397 (68)			44
Would you co-manage a patient with an osteopath (if clinically appropriate)?	193 (33)	393 (67)	1 (−4–3)	0.70	44
Are you aware that all primary chiropractic education in Australia is university based?	456 (78)	126 (22)			48
Are you aware that all primary osteopathic education in Australia is university based?	313 (54)	269 (46)	24 (21–28)	< 0.01*	48
Are you aware that primary chiropractic education in Australia is a 5 year full-time program?	379 (65)	202 (35)			49
Are you aware that primary osteopathic education in Australia is a 5 year full-time program?	265 (46)	312 (54)	19 (16–23)	< 0.01*	53
Would you be interested in learning more about primary chiropractic education in Australia?	185 (32)	388 (68)			57
Would you be interested in learning more about primary osteopathic education in Australia?	209 (37)	359 (63)	5 (2–7)	< 0.01*	62

*CI* Confidence interval, *O* Osteopathic, *C* Chiropractic

* *p* < 0.05

^a^ All figures given as n (%) of total responses to that question except for non-responders

^b^ Responses recorded as ‘Maybe’, ‘Don’t remember’ and ‘Don’t know’ have been included under the NO column for statistical analysis

^c^ Difference (%) in YES answers for pairs of questions calculated using McNemar’s test for dependent binary data

^d^ Figures given as number of non-responders

**Table 3:** Difference in GP attitudes about Chiropractors and Osteopaths: ordinal response questions^a^ (*n* = 630)

Survey question	Strongly agree	Agree	Total^b^	Neither agree nor disagree	Disagree	Strongly disagree	Total^c^	Difference^d^ (95 % CI)	p		Non-responders^i^
To what extent do you agree with the following statement: “Chiropractic treatment could benefit some of my patients.”	43 (7)	167 (28)	210 (35)	146 (24)	139 (23)	112 (19)	397 (65)				23
To what extent do you agree with the following statement: “Osteopathic treatment could benefit some of my patients.”	46 (7)	160 (26)	206 (34)	201 (32)	107 (17)	97 (16)	405 (66)	−1 (−3–5)	0.74		19
To what extent do you agree with the following statement: “They (chiropractors) have provided useful and/or meaningful correspondence with me about co-management of patients.”	16 (4)	63 (14)	79 (18)	74 (17)	138 (31)	156 (34)	368 (82)				183
To what extent do you agree with the following statement: “They (osteopaths) have provided useful and/or meaningful correspondence with me about co-management of patients.”	19 (7)	50 (17)	69 (24)	68 (24)	62 (21)	90 (31)	220 (76)	−6 (−13– –4)	< 0.01*		341
To what extent do you agree with the following statement: “I would like to have a better professional relationship with the local chiropractor(s).”	26 (5)	107 (18)	133 (23)	213 (36)	143 (24)	104 (17)	460 (77)				37
To what extent do you agree with the following statement: “I would like to have a better professional relationship with the local osteopath(s).”	28 (5)	117 (20)	145 (25)	236 (40)	116 (19)	95 (16)	447 (75)	−2 (−5–1)	0.18		38
To what extent do you agree with the following statement: “The ongoing media coverage surrounding chiropractic has made it difficult to understand the role of the chiropractor in health care.”	39 (7)	125 (22)	164 (29)	211 (37)	137 (23)	65 (11)	413 (71)				53
To what extent do you agree with the following statement: “The ongoing media coverage surrounding osteopathy has made it difficult to understand the role of the osteopath in health care.”	23 (4)	62 (11)	85 (15)	301 (52)	138 (23)	57 (10)	496 (85)	14 (11–17)	< 0.01*		49
To what extent do you agree with the following statement: “There is a growing body of evidence available on the efficacy of chiropractic treatment.”	19 (3)	41 (7)	60 (10)	140 (24)	189 (33)	185 (33)	514 (90)				56
To what extent do you agree with the following statement: “There is a growing body of evidence available on the efficacy of osteopathic treatment.”	9 (2)	56 (9)	65 (11)	228 (40)	141 (24)	143 (25)	512 (89)	−1 (−3–2)	0.67		53
To what extent do you agree with the following statement: “Chiropractic education in Australia is primarily evidence based.”	9 (2)	34 (6)	43 (8)	133 (23)	165 (29)	236 (41)	534 (93)				53
To what extent do you agree with the following statement: “Osteopathic education in Australia is primarily evidence based.”	5 (1)	51 (9)	56 (10)	233 (40)	116 (20)	173 (30)	522 (90)	−2 (−5–1)	0.04*		52
	Very know-ledgeable	Know-ledgeable	Some knowledge	Total^e^	Not very much knowledge	No knowledge	Total^f^	Difference^d^ (95 % CI)	p		Non-responders^i^
How would you rate your knowledge of chiropractic and its mechanism of treatment?	52 (9)	149 (24)	261 (43)	462 (76)	127 (21)	18 (3)	145 (24)				23
How would you rate your knowledge of osteopathy and its mechanism of treatment?	33 (5)	87 (14)	221 (36)	341 (56)	199 (32.6)	70 (11.5)	269 (44)	20 (16–24)	< 0.01*		20
	Very satisfied	Satisfied	Total^g^	Neither satisfied nor dissatisfied	Dissatisfied	Very dissatisfied	Haven’t communicated enough to respond	Total^h^	Difference^d^ (95 % CI)	p	Non-responders^i^
Are you satisfied with the methods of communication between chiropractors and doctors?	13 (2)	35 (6)	48 (8)	118 (20)	97 (16)	88 (15)	242 (41)	545 (92)			37
Are you satisfied with the methods of communication between osteopaths and doctors?	11 (2)	42 (7)	53 (9)	132 (22)	48 (8)	58 (10)	301 (51)	539 (91)	−1 (−3–2)	0.49	38

*CI* Confidence interval

* *p* < 0.05

^a^ All figures given as n (%) of total responses to that question except for non-responders

^b^ Total responses recorded as ‘Strongly agree’ and ‘Agree’

^c^ Total responses recorded as ‘Neither agree nor disagree’, ‘Disagree’ and ‘Strongly disagree’

^d^ Difference (%) in Total^b^, Total^e^ or Total^g^ answers respectively for pairs of questions calculated using McNemar’s test for dependent binary data

^e^ Total response recorded as ‘Very knowledgeable’, ‘Knowledgeable’ and ‘Some knowledge’

^f^ Total responses recorded as ‘Not very much knowledge’ and ‘No knowledge’

^g^ Total responses recorded as ‘Very satisfied’ and ‘Satisfied’

^h^ Total responses recorded as ‘Neither satisfied nor dissatisfied’, ‘Dissatisfied’, ‘Very dissatisfied’ and ‘Haven’t communicated enough to respond’

^i^ Figures given as number of non-responders

When asked if they were satisfied with the methods of communication between professions, 31 % of GPs were either dissatisfied or very dissatisfied with the methods of communication between themselves and chiropractors while 41 % hadn’t had enough communication to be able to respond to the question. Similarly, 18 % of GPs were either dissatisfied or very dissatisfied with the methods of communication between themselves and osteopaths while 51 % hadn’t had enough communication to be able to respond to the question. As for referrals to GPs, 49 % reported having a patient referred to them by a chiropractor while only 30 % reported referral by an osteopath. Approximately two-thirds of GPs reported not being interested in learning more about the education of chiropractors or osteopaths (68 % and 63 % respectively).

The top three conditions that GPs thought chiropractic or osteopathic treatment could be helpful for were the same for both professions: mechanical low back pain, neck and upper back pain due to muscle tension and cervicogenic headache.

The proportion of non-responders ranged from 3 to 10 %, except for the questions related to whether chiropractors and osteopaths had provided meaningful correspondence about patient co-management where the non-response rate increased to 29 % for chiropractors and 54 % for osteopaths.

Comments in response to the open-ended and mixed questions revealed some common themes for both professions. For chiropractic, the themes centred on the lack of evidence-base, potential harm to patients, exploitative nature of some practices and lack of trust towards the profession. For osteopathy, the themes centred on a lack of evidence-base, potential harm to patients and a lack of trust towards the profession as well as comments showing that many GPs were unaware of what osteopaths did.

## Discussion

As far as we can ascertain, this is the first attempt to assess the national views of Australian GPs towards chiropractors and osteopaths since 2005. Previous studies have found that GPs' views were different for different CM therapies. For example, Cohen et al. found that GPs considered non-medicinal and non-manipulative therapies like acupuncture, massage and hypnosis to be effective and safe whereas other therapies like aromatherapy and reflexology were considered relatively ineffective but safe [2]. These differing attitudes were reflected in GP referral rates: 87 % for massage, 83 % for acupuncture, 65 % for meditation, 63 % for osteopathy and 60 % for chiropractic. Results from our respondents suggest that there has been a shift in views towards chiropractic and osteopathy. If these results are confirmed in a larger-scale survey they would be in clear contrast to the situation that existed in 2005 where the attitudes of Australian GPs towards CM were considered to have “not changed appreciably” in the preceding 7 years [2].

Results from our respondents suggest that the views of Australian GPs towards chiropractic and osteopathy are changing towards a more negative view of the professions and their practices. Our results also suggest that many GPs may have become intolerant to learning more about these commonly used types of CM. The results also appear to suggest that GPs have differing views about each of the professions. This may simply be a product of there being less osteopaths in Australia and therefore less opportunities for GPs to become familiar with the profession.

If the trend towards a more negative view is confirmed as an accurate appraisal of attitudes in the broader GP community then the ‘highly emotive’ comments about the legitimacy of CM made by senior doctors and scientists [33] may be influencing the way GPs discuss CM therapies in general with their patients. As healthcare systems become increasingly patient-centred any impact on doctor-patient communication on issues such as the use of chiropractic and osteopathy which are often patient-driven needs to be carefully scrutinised. Such a situation would have the potential to set back relations between the professions. The Australian Health Practitioner Regulation Agency (AHPRA) is the national organisation responsible for implementing the Australian National Registration and Accreditation Scheme [30]. The scheme covers a range of health professions including medical practitioners, chiropractors and osteopaths. It promotes cross-professional consultation and collaboration in managing common regulatory issues such as accreditation, research and workforce reform [34]. If one profession becomes intolerant towards the practices of another it could lead to unwillingness to work together on common matters. If the views described in this cross-sectional study were to be confirmed as representative of those held by the broader GP community then the chiropractic and osteopathic professions would need to identify the issues that led to the change and begin to put in place measures that address these issues. This would include strengthening and promoting the evidence-base of these professions.

It is possible that the differences in demographics between our respondents and the national average could have influenced the results. However, there is no way of measuring the impact of individual elements such as age or years in practice on responses to online surveys of this kind.

It is important to consider other possible reasons why the views have deteriorated including whether attitudes differ towards management of musculoskeletal conditions compared to non-musculoskeletal conditions. For example, the changes in attitude may simply reflect the availability of relevant information in medical journals or may be the product of different levels of coverage of each profession in the Australian medical media. As mentioned previously, it may be that GPs are less informed about osteopathy compared to chiropractic. It is also possible that GPs hold very different views towards chiropractors and osteopaths with respect to their management of musculoskeletal and non-musculoskeletal conditions where the evidence base for the two is quite different [12–15].

### Limitations

While we consider the views being reported here as important enough to warrant publication in their own right, we acknowledge the inherent limitations associated with analysing cross-sectional data in this manner. These limitations include generalisability and selection bias. With respect to generalisability, our 630 responses represent 2.6 % of the total number of vocationally registered GPs. While this may be adequate for a cross-sectional study, any generalisations inferred from the data should be qualified. Notwithstanding, the size of our sample is comparable to the 2005 study which reported results based on a response rate of 3.4 % [2]. Despite the differences described previously, we believe our cohort is a reasonable representation of the national body of vocationally registered GPs. As for selection bias, we acknowledge that it is possible that the self-selection process used in this cross-sectional study meant that more GPs with strong negative views about chiropractic and/or osteopathy completed the survey. Even if this were the case it should not detract from the finding that there is a growing number of GPs who are clearly dissatisfied with the practice of chiropractors and osteopaths in Australia. A limitation in any study of this kind is the potential for outside factors to influence the views of respondents at the time of completing the survey. In this case, it is worth noting that there was an increase in the level of negative press about chiropractic in Australia in the lead up to and during the survey period [26].

## Conclusions

The results of this study suggest that there has been a negative shift in the views of GPs who completed the survey towards chiropractic and osteopathy. If confirmed in a larger-scale study then there may be implications for GP-patient communication. Strongly held negative views about chiropractic and osteopathy have the potential to influence discussions with patients about their treatment choices. Given the increasing demand for chiropractic and osteopathic services, GPs may need to increase their understanding of chiropractic and osteopathy if they are to satisfy the requirements of a healthcare system that is becoming increasingly patient-centred.

The results from this cross-sectional study suggest that obtaining representative GP views using online surveys is difficult and another approach (e.g. semi-structured interviews) is needed to supplement or replace the current recruitment strategy.

## Additional files

Additional file 1: SURVEY QUESTIONNAIRE. (PDF 350 kb)
Additional file 2: Responses to a National Survey on the views of General Practitioners (GPs) towards the Chiropractic and Osteopathic professions. (XLS 223 kb)

## Abbreviations

AHPRA: Australian Health Practitioner Regulation Agency
CM: Complementary Medicine
GP: General Practitioner
RACGP: Royal Australian College of General Practitioners
