# Supplementary material for: An indication of current views of Australian general practitioners towards chiropractic and osteopathy: a cross-sectional study
Source: Chiropr Man Therap. 2016 Nov 1;24:37. doi: 10.1186/s12998-016-0119-6 (PMC5088656; doi:10.1186/s12998-016-0119-6)
Supplement: Additional file 1: — SURVEY QUESTIONNAIRE. (PDF 350 kb) [file 12998_2016_119_MOESM1_ESM.pdf]

Department of Chiropractic
Faculty of Science
MACQUARIE UNIVERSITY NSW 2109
Phone: +61 (0)2 98506392
Fax: +61 (0)2 98509389
Email: robyn.beirman@mq.edu.au

PARTICIPANT INFORMATION SHEET

You are invited to participate in an anonymous survey of the perceptions general medical practitioners (GPs) have regarding chiropractic and osteopathy. The purpose of the study is to provide insight into the knowledge and views of Australian GPs about these professions.
This study is being conducted by Dr. Robyn Beirman (Macquarie University), Dr Roger Engel (Macquarie University) and Dr Sandra Grace (Southern Cross University). Any questions or concerns regarding this study can be forwarded to Dr Beirman, who can be contacted on (02) 9850 6392 or robyn.beirman@mq.edu.au.
If you decide to participate, you will be asked to complete the survey, which should take about 10 minutes of your time.
The survey will query your perceptions and knowledge of chiropractic and osteopathy in Australia, through a mix of objective and open-ended questions.
Any information or personal details gathered in the course of the study will remain confidential, except as required by law. No individual will be identified in any publication of the results.
The only people with access to the data are the investigators named above.
A summary of the results of the data will be made available to the RACGP.
Participation in this study is entirely voluntary: you are not obliged to participate and if you decide to participate, you are free to withdraw at any time without having to give a reason and without consequence. Consent will be assumed to be given by submission of the completed survey.
The ethical aspects of this study have been approved by the Macquarie University Human Research Ethics Committee. If you have any complaints or reservations about any ethical aspect of your participation in this research, you may contact the Committee through the Director, Research Ethics (telephone (02) 9850 7854; email ethics@mq.edu.au). Any complaint you make will be treated in confidence and investigated, and you will be informed of the outcome.

A NATIONAL SURVEY ON THE VIEWS OF GENERAL PRACTITIONERS TOWARDS THE CHIROPRACTIC AND OSTEOPATHIC PROFESSIONS

Q1. How long have you been a medical practitioner in general practice?

0-2 years ☐ 3-5 years ☐ 6-10 years ☐ 11-15 years ☐ 16-20 years ☐ 20+ years ☐

Q2. What is the postcode of your principle practice? _______________

Q3. Are you male or female? Male ☐ Female ☐

Q4. What is your age? Under 30 ☐ 30-40 ☐ 41-50 ☐ 51-60 ☐ Over 60 ☐

Q5. Where do you get MOST of your information regarding Chiropractic? (Indicate ONE only)

Medical journals (peer-reviewed) ☐ Non-medical journals (peer-reviewed) ☐

Popular media ☐ Chiropractors themselves ☐ Medical associations ☐

Don't really get much information regarding it ☐

Other:____

Q6. Where do you get MOST of your information regarding Osteopathy? (Indicate ONE only)

Medical journals (peer-reviewed) ☐ Non-medical journals (peer-reviewed) ☐

Popular media ☐ Osteopaths themselves ☐ Medical associations ☐

Don't really get much information regarding it ☐

Other:____

Q7. Have you ever *witnessed* a treatment by a chiropractor? Yes ☐ No ☐

Q8. Have you ever *witnessed* a treatment by an osteopath? Yes ☐ No ☐

Q9. Have you ever been a *recipient* of a treatment by a chiropractor? Yes ☐ No ☐

Q10. Have you ever been a *recipient* of a treatment by an osteopath? Yes ☐ No ☐

Q11. How would you rate your knowledge of chiropractic and its mechanism of treatment?

Very knowledgeable ☐ Knowledgeable ☐ Some knowledge ☐

Not very much knowledge ☐ No knowledge ☐

Q12. How would you rate your knowledge of osteopathy and its mechanism of treatment?

Very knowledgeable ☐ Knowledgeable ☐ Some knowledge ☐

Not very much knowledge ☐ No knowledge ☐

Q13. To what extent do you agree with the following statement?

"Chiropractic treatment could benefit some of my patients"

Strongly agree ☐ Agree ☐ Neither agree nor disagree ☐ Disagree ☐ Strongly disagree ☐

Q14. To what extent do you agree with the following statement?

"Osteopathic treatment could benefit some of my patients"

Strongly agree ☐ Agree ☐ Neither agree nor disagree ☐ Disagree ☐ Strongly disagree ☐

Q15. Have *you* ever referred a patient to a chiropractor?

Yes ☐ Please go to question 17

No ☐ If no, please give your reason

____

____

Q16. Would a better understanding of chiropractic make you reconsider referral?

Yes ☐ No ☐ Maybe ☐

Q17. Have *you* ever referred a patient to an osteopath?

Yes ☐ Please go to question 19

No ☐ If no, please give your reason

____

____

Q18. Would a better understanding of osteopathy make you reconsider referral? Yes ☐ No ☐ Maybe ☐

Q19. Have you ever had a patient *referred to you* by a chiropractor? Yes ☐ No ☐ Don't remember☐

Q20. Have you ever had a patient *referred to you* by an osteopath? Yes ☐ No ☐ Don't remember☐

Q21. Do you know any of the chiropractors in the area around your practice?

Yes ☐ Please go to question 22

No ☐ Please go to question 23

Q22. To what extent do you agree with the following statement: "They have provided useful and/or meaningful correspondence with me about co-management of patients"

Strongly agree ☐ Agree ☐ Neither agree nor disagree ☐ Disagree ☐ Strongly disagree ☐

Q23. To what extent do you agree with the following statement: "I would like to have a better professional relationship with the local chiropractor(s)"

Strongly agree ☐ Agree ☐ Neither agree nor disagree ☐ Disagree ☐ Strongly disagree ☐

Q24. Do you know any of the osteopaths in the area around your practice?

Yes ☐ Please go to question 25

No ☐ Please go to question 26

Q25. To what extent do you agree with the following statement: "They have provided useful and/or meaningful correspondence with me about co-management of patients"

Strongly agree ☐ Agree ☐ Neither agree nor disagree ☐ Disagree ☐ Strongly disagree ☐

Q26. To what extent do you agree with the following statement: "I would like to have a better professional relationship with the local osteopath(s)."

Strongly agree ☐ Agree ☐ Neither agree nor disagree ☐ Disagree ☐ Strongly disagree ☐

Q27. Are you satisfied with the methods of communication between chiropractors and doctors?

Very satisfied ☐ Satisfied ☐ Neither satisfied nor dissatisfied ☐ Dissatisfied ☐ Very dissatisfied ☐

Haven't communicated or communicated often enough to respond ☐

Any comments?

____

____

Q28. Are you satisfied with the methods of communication between osteopaths and doctors?

Very satisfied ☐ Satisfied ☐ Neither satisfied nor dissatisfied ☐ Dissatisfied ☐ Very dissatisfied ☐

Haven't communicated or communicated often enough to respond ☐

Any comments?

____

____

Q29. For which of the following conditions do you believe that chiropractic treatment can be helpful?

Tension headache	yes☐ no☐ I don’t know☐
Cervicogenic headache	yes☐ no☐ I don’t know☐
Migraine	yes☐ no☐ I don’t know☐
Mechanical low back pain	yes☐ no☐ I don’t know☐
Facilitating mobility in patients with arthritides	yes☐ no☐ I don’t know☐
Neck and upper back pain due to muscle tension	yes☐ no☐ I don’t know☐

Q30. For which of the following conditions do you believe that osteopathic treatment can be helpful?

Tension headache	yes☐ no☐ I don’t know☐
Cervicogenic headache	yes☐ no☐ I don’t know☐
Migraine	yes☐ no☐ I don’t know☐
Mechanical low back pain	yes☐ no☐ I don’t know☐
Facilitating mobility in patients with arthritides	yes☐ no☐ I don’t know☐
Neck and upper back pain due to muscle tension	yes☐ no☐ I don’t know☐

Q31. Would you co-manage a patient with a chiropractor (if clinically appropriate)?

Yes ☐ No ☐ Maybe ☐

Q32. Would you co-manage a patient with an osteopath (if clinically appropriate)?

Yes ☐ No ☐ Maybe ☐

Q33. To what extent do you agree with the following statement?

"The ongoing media coverage surrounding chiropractic has made it difficult to understand the role of the chiropractor in health care".

Strongly agree ☐ Agree ☐ Neither agree nor disagree ☐ Disagree ☐ Strongly disagree ☐

Q34. To what extent do you agree with the following statement?

"The ongoing media coverage surrounding osteopathy has made it difficult to understand the role of the osteopath in health care".

Strongly agree ☐ Agree ☐ Neither agree nor disagree ☐ Disagree ☐ Strongly disagree ☐

Q35. To what extent do you agree with the following statement?

"There is a growing body of evidence available on the efficacy of chiropractic treatment"

Strongly agree ☐ Agree ☐ Neither agree nor disagree ☐ Disagree ☐ Strongly disagree ☐

Q36. To what extent do you agree with the following statement?

"There is a growing body of evidence available on the efficacy of osteopathic treatment"

Strongly agree ☐ Agree ☐ Neither agree nor disagree ☐ Disagree ☐ Strongly disagree ☐

Q37. To what extent do you agree with the following statement?

"Chiropractic education in Australia is primarily evidence based"

Strongly agree ☐ Agree ☐ Neither agree nor disagree ☐ Disagree ☐ Strongly disagree ☐

Q38. To what extent do you agree with the following statement?

"Osteopathic education in Australia is primarily evidence based"

Strongly agree ☐ Agree ☐ Neither agree nor disagree ☐ Disagree ☐ Strongly disagree ☐

Q39. Are you aware that

a. All primary chiropractic education in Australia is university based Yes☐ No☐

b. Primary chiropractic education in Australia is a 5 year full-time program Yes☐ No☐

c. All primary osteopathic education in Australia is university based Yes☐ No☐

d. Primary osteopathic education in Australia is a 5 year full-time program Yes☐ No☐

Q40. Would you be interested in learning more about primary chiropractic education in Australia? Yes☐ No☐

Q41. Would you be interested in learning more about primary osteopathic education in Australia? Yes☐ No☐

Q42. Please add any comments about your attitudes to either the Chiropractic profession or Chiropractic education

Q43. Please add any comments about your attitudes to either the Osteopathic profession or Osteopathic education
